# Supplementary material for: High power density redox-mediated Shewanella microbial flow fuel cells
Source: Nat Commun. 2024 Sep 27;15:8302. doi: 10.1038/s41467-024-52498-w (PMC11448506; doi:10.1038/s41467-024-52498-w)
Supplement: Supplementary file 1 — Supplementary Information [file 41467_2024_52498_MOESM1_ESM.pdf]

Supplementary Information

High power density redox-mediated *Shewanella* microbial flow fuel cells

Leyuan Zhang[1,2], Yucheng Zhang[1], Yang Liu[1], Sibo Wang[2], Calvin K. Lee[3], Yu Huang[1,4]*, Xiangfeng Duan[2,4]*

[1]Department of Materials Science and Engineering, University of California, Los Angeles, Los Angeles, CA 90095, USA. [2]Department of Chemistry and Biochemistry, University of California, Los Angeles, Los Angeles, CA 90095, USA. [3]Department of Bioengineering, University of California, Los Angeles, Los Angeles, CA 90095, USA. [4]NanoSystems Institute, University of California, Los Angeles, Los Angeles, CA 90095, USA.

**Correspondence to Y.H. and X.D.: yhuang@seas.ucla.edu; xduan@chem.ucla.edu*

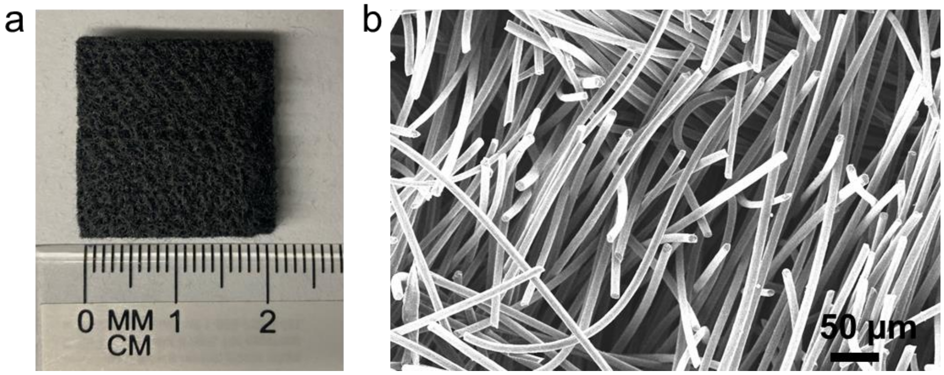

Supplementary Figure 1 | Physical characterization of carbon felt. a, Optical photo of carbon felt as current collectors. **b**, Scanning electron microscope (SEM) picture of carbon felt.

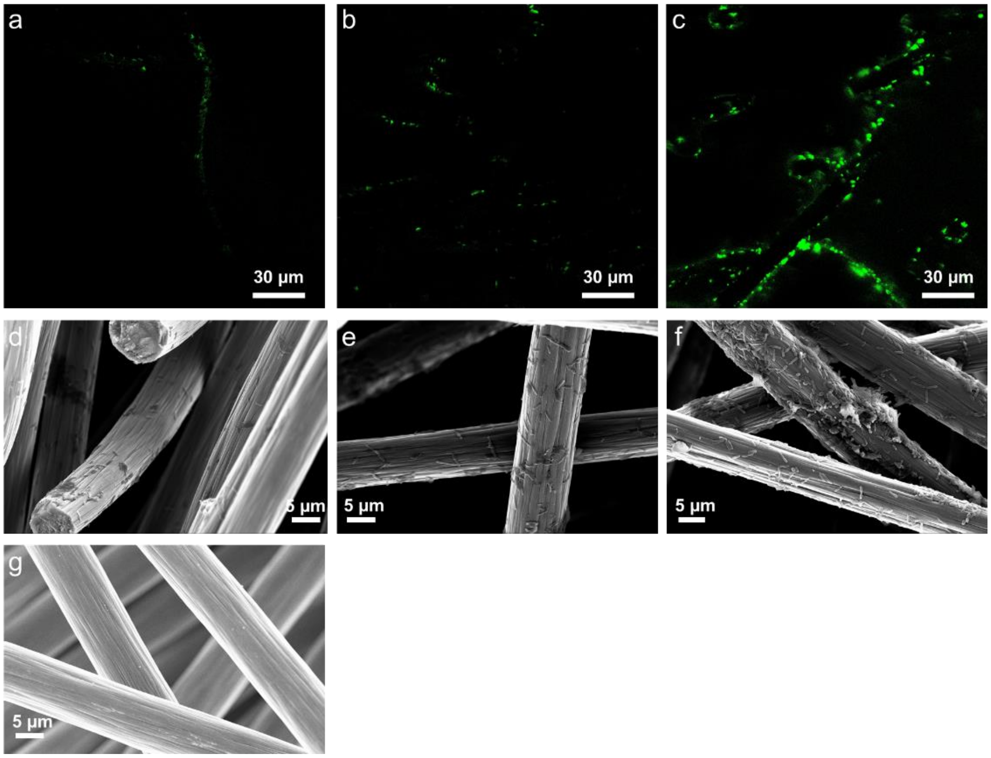

Supplementary Figure 2 | Characterization of bacteria within carbon felts after flowing for various time. a-c, Confocal laser scanning microscopy (CLSM) images of the *Shewanella oneidensis* (*S. oneidensis*) bacteria inside carbon felts after continuously flowing for (**a**) 10 minutes, (**b**) 6 hours, and (**c**) 24 hours. **d-f**, SEM images of the *S. oneidensis* bacteria inside carbon felts after continuously flowing for (**d**) 10 minutes, (**e**) 6 hours, and (**f**) 24 hours. **g**, SEM image of the nascent carbon felt before the flowing.

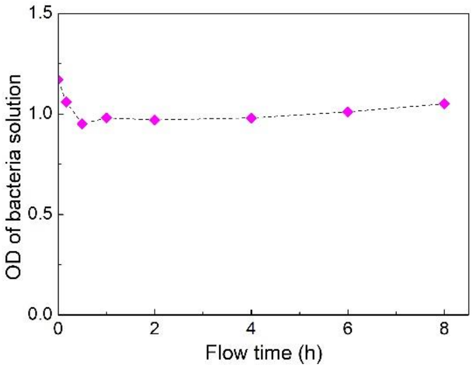

Supplementary Figure 3 | The OD change of the bacteria anolyte in MFFCs during the continuous flowing. The OD showed a slight decrease of (~0.2) in the first ~30 mins and remained stable with a slow growth, indicating the major power source is from the flowing bacteria.

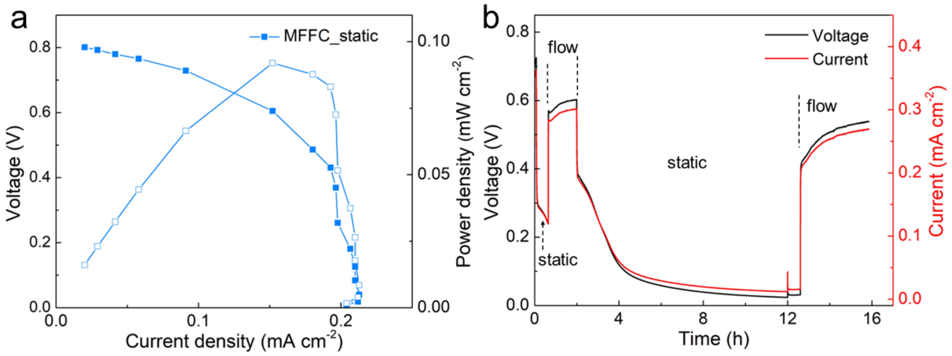

Supplementary Figure 4 | Electrochemical characterization of the MFFC at static state. a, Polarization curves and power output of the MFFC with stopping the flow measured with different external resistors. **b**, Voltage output of the MFFC with carbon felt with a 500 Ω external resistor when stopping or flowing the anolyte and catholyte. At the static state, the MFFC (without redox mediators) shows much lower current and power output (0.21 mA cm^{-2}, 0.092 mW cm^{-2}) compared to the flowing MFFC (0.7 mA cm^{-2}, 0.19 mW cm^{-2}). The flow dynamic studies further show that the output voltage immediately drops from 0.60 V to 0.38 V when the flow is stopped, which gradually decreases to nearly 0 V in 4 hours, which can be attributed to the diffusion limitations (without active flow, the lactate near carbon felt electrode relies on diffusion for replenishing, and can be depleted overtime). When the flow is resumed, the output voltage quickly recovers again.

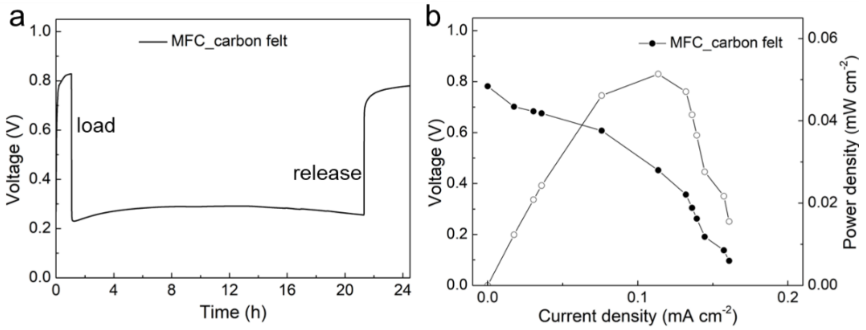

Supplementary Figure 5 | Electrochemical characterization of the MFCs with carbon felt anodes. a, Open circuit voltage and voltage output of H-type MFCs with carbon felt with a 500 Ω external resistor. **b**, Polarization curves and power output of H-type MFCs with carbon felt electrode measured with different external resistors.

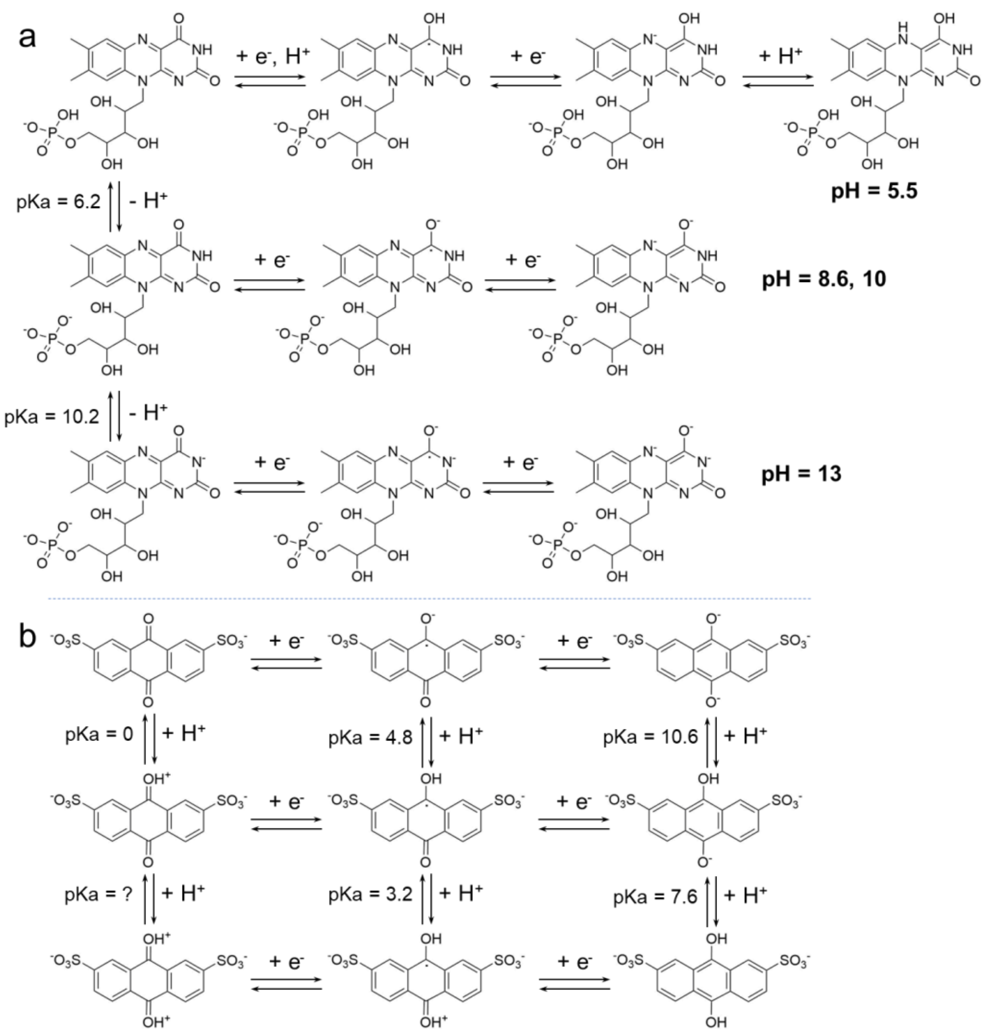

Supplementary Figure 6 | Redox reaction and proton transfer process of (a) FMN-Na and (b) 2,7-AQDS. In the electron transfer from bacteria to redox mediators, it could be coupled with proton transfer to keep electroneutrality of solution, which can cause the pH variation. Correspondingly, the redox potential of redox mediators can also vary as it is pH sensitive. For the FMN-Na mediator, there is the deprotonation process with the increase of pH values (0.7<pH<6.2, 6.2<pH<10.2, pH>10.2), and the reaction mechanism is also related to pH. In the pH range of 8.6-10.0, the redox reaction does not directly involve proton transfer. For the 2,7-AQDS mediator, similarly, the potential and reaction mechanism are also dependent on the pH environment. At high pH (>12), the reaction is a simple EE process. With pH decreasing, the protonation changes the potential and reaction pathway. At pH 10, the EECC process dominates. In the pH range from 4 to 7, it changes to an ECEC process, and a CECE mechanism occurs at low pH (<1). These two schemes of squares framework are summarized from the reported data[3,4].

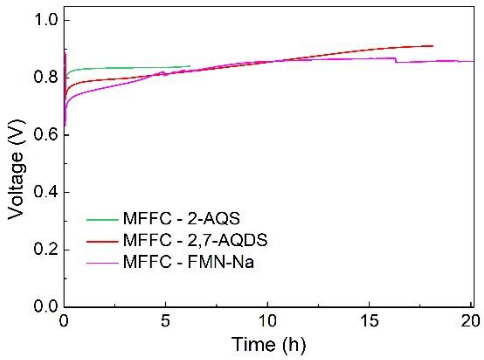

Supplementary Figure 7 | Open circuit voltage (OCV) of the MFFCs with redox mediators. OCVs of MFFCs with various redox mediators in the extended time scale.

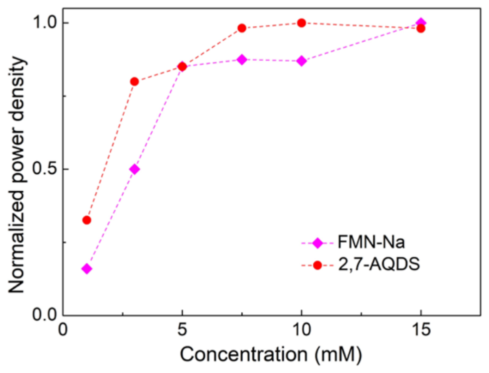

Supplementary Figure 8 | The power density of redox-mediated MFFCs at various concentrations of redox mediators.

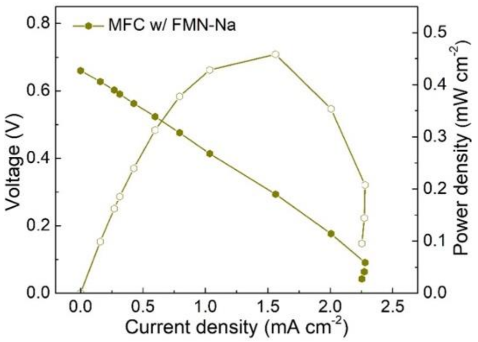

Supplementary Figure 9 | Power density of MFCs with redox mediators. Polarization curves and power output of conventional MFCs with the FMN-Na mediator measured with external resistors.

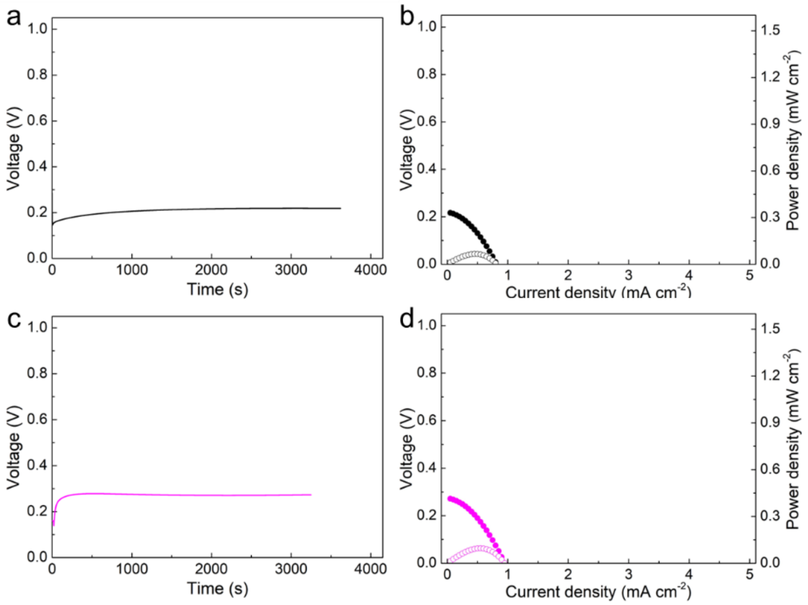

Supplementary Figure 10 | Electrochemical characterizations of the MFFC system without bacteria. a, Open circuit voltage of the MFFC with pure medium without *S. oneidensis* bacteria. **b**, Instant power polarization curve of the MFFC with pure medium without *S. oneidensis* bacteria. **c**, Open circuit voltage of the MFFC with adding 20 mM FMN-Na into pure medium without *S. oneidensis* bacteria. **d**, Instant power polarization curve of the MFFC with adding 20 mM FMN-Na into pure medium without *S. oneidensis* bacteria.

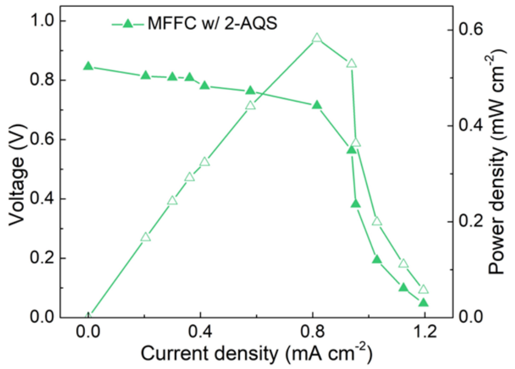

Supplementary Figure 11 | Power density of MFFCs with redox mediators. Polarization curves and power output of MFFCs with the 2-AQS redox mediator measured with external resistors.

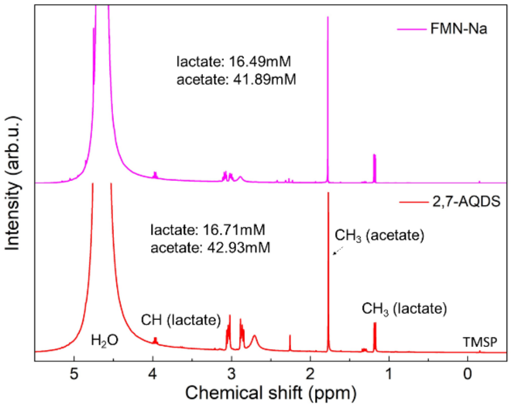

Supplementary Figure 12 | Lactate and acetate concentration in the bacteria medium after operation. ^{1}H NMR spectroscopy of the bacteria anolyte with removing bacteria after the long-term test of MFFCs with the FMN-Na or 2,7-AQDS mediators. According to the concentration of lactate and acetate retained in the medium solution, it confirms that the *S. oneidensis* bacteria utilize the lactate oxidization with a four-electron transfer reaction. Note that as small amount of solution retained during the centrifugation process of bacteria culture before the test, the overall concentration is slightly less than 60 mM.

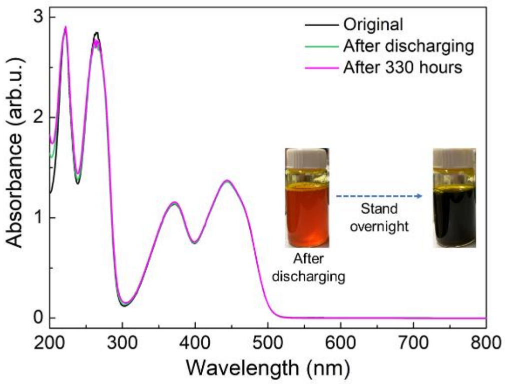

Supplementary Figure 13 | Chemical stability and biocompatibility of the redox mediators with *S. oneidensis* bacteria. UV-vis spectroscopy of bacteria solution with FMN-Na at different states. For the UV-vis measurement, the target solution was diluted about 200 times in DI-water. The black curve is the UV-vis spectroscopy of solution at the original state; The green curve is the UV-vis spectroscopy of solution after discharging with an external resistor for a long time (~15 h); The magenta curve is the UV-vis spectroscopy of solution after storing for about 330 hours. The inset are the photos of bacteria solution with FMN-Na after discharging and standing overnight. The color change of bacteria solution observed after standing for a period of time indicates the reduction of FMN-Na, which means the bacteria are still active.

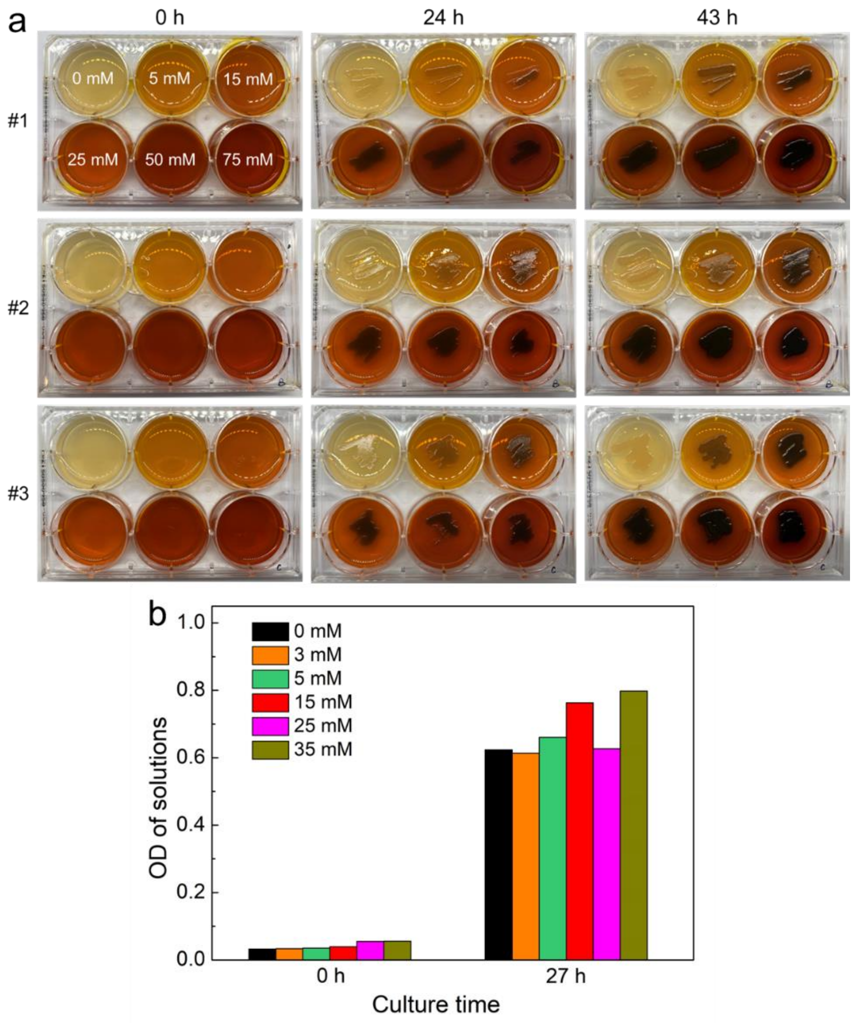

Supplementary Figure 14 | Antimicrobial activity of the FMN-Na mediator against *S. oneidensis*. a, The bacteria incubation on the LB-Agar plate with various concentrations of redox mediators. **b**, The bacteria culture in the lactate medium with various concentrations of redox mediators. The used FMN-Na mediator should be compatible with *S. oneidensis*, as the electrochemical operation is reasonable. Here we further investigated the antimicrobial activity of the FMN-Na mediator against *S. oneidensis*. In particular, we tried to determine the minimum inhibitory concentration via inoculating the bacteria on the LB-Agar plate with different concentrations of FMM-Na (0, 5, 15, 25, 50, and 75 mM). *S. oneidensis* can grow under all these conditions without obvious difference, suggesting the minimum inhibitory concentration of FMN-Na is higher than 75 mM. Because the used concentration of redox mediators is ~3-25 mM (<<75 mM), the antimicrobial activity of FMN-Na is largely negligible under our operation condition. In addition, we also studied the antimicrobial activity of FMN-Na by culturing the bacteria in the lactate medium with different concentrations (0, 3, 5, 15, 25, 35 mM). As the saturated concentration of FMN-Na in the lactate medium is about 35 mM, we did not use higher concentrations for this method. It also showed that *S. oneidensis* could grow similarly in the lactate medium with or without the FMN-Na mediator.

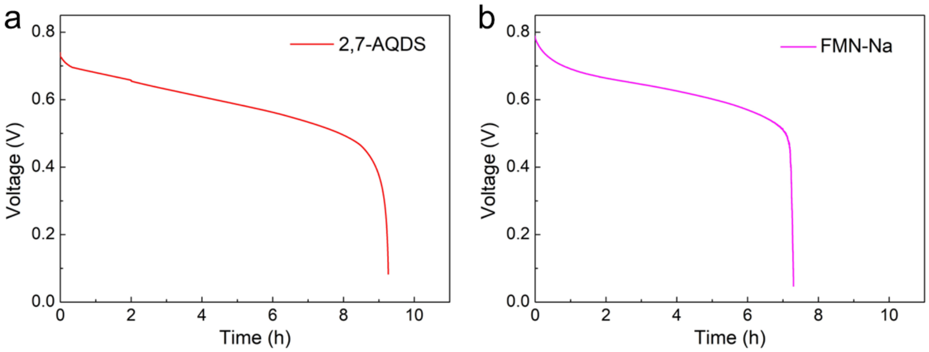

Supplementary Figure 15 | Long-term operation of the redox-mediated MFFCs with the Nafion membrane. a, Long-term operation of the 2,7-AQDS-mediated MFFCs at a constant current of 2.5 mA cm^{-2}. **b**, Long-term operation of the FMN-Na-mediated MFFCs at a constant current of 2.5 mA cm^{-2}.

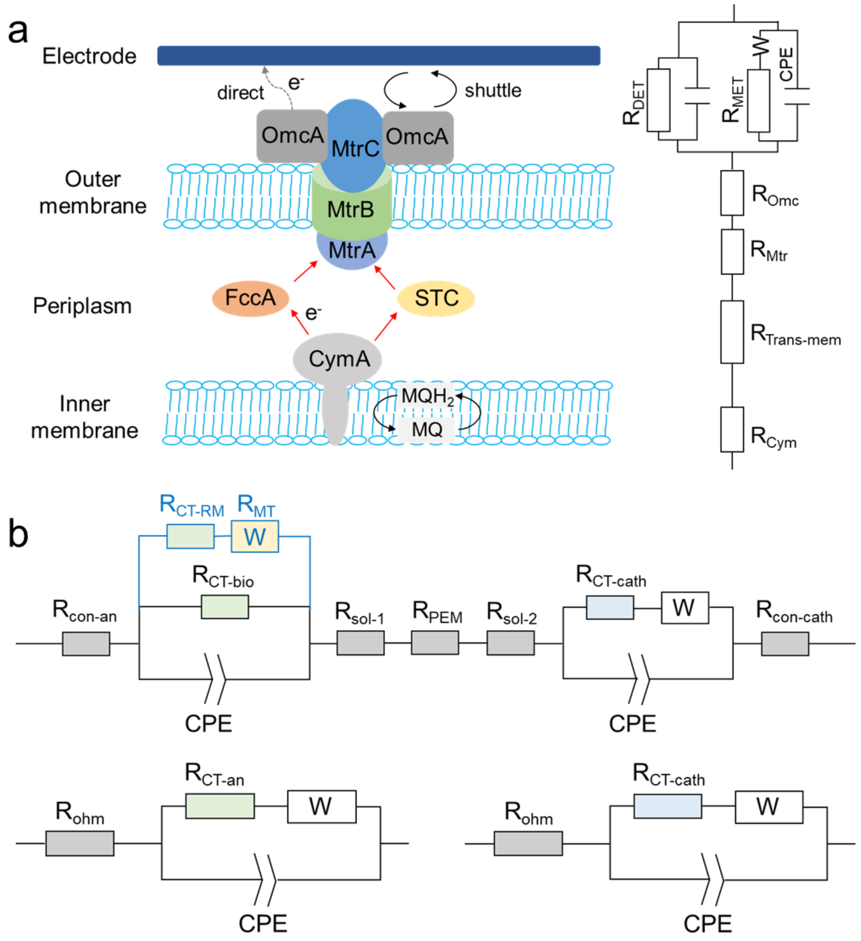

Supplementary Figure 16 | Electron transfer chain of *S. oneidensis* and equivalent circuit model for internal resistance analysis of the MFFC system. a, The metabolic electrons are transferred from cytoplasm to outer membrane by hopping through specific cytochromes including CymA, FccA & STC, MtrABC, and OmcA, represented as the resistance, R_{Cym}, $R_{\mathrm{Trans\text{-}mem}}$, R_{Mtr}, and R_{Omc} in the equivalent circuits, respectively. The extracellular electron transfer (EET) process includes two parallel paths: direct electron transfer (R_{DET}), and indirect redox shuttling process (mediated electron transfer, R_{MET}). **b**, The equivalent circuit of microbial flow fuel cell (MFFC) and the separate anode or cathode reaction, respectively. The $R_{\mathrm{con\text{-}an}}$ and $R_{\mathrm{con\text{-}cath}}$ represent the contact resistance of anode and cathode. R_{sol} and R_{PEM} are the solution resistance and proton exchange membrane resistance, respectively. $R_{\mathrm{CT\text{-}bio}}$ corresponds to the extracellular electron transfer (EET) between *S. oneidensis* and electrode. $R_{\mathrm{CT\text{-}RM}}$ is related to the charge transfer resistance with redox mediators, and R_{MT} is the mass transport resistance. $R_{\mathrm{CT\text{-}cath}}$ is the charge transfer resistance of the cathode reaction. $R_{\mathrm{CT\text{-}an}}$ is the charge transfer resistance of the anode reaction (e.g., $R_{\mathrm{CT\text{-}bio}}$ or $R_{\mathrm{CT\text{-}RM}}$). The separate reaction resistance is based on the three-electrode system. The R_{ohm} is the sum of the Ohmic resistance of the separate anode or cathode reaction, including the contact and solution resistance.

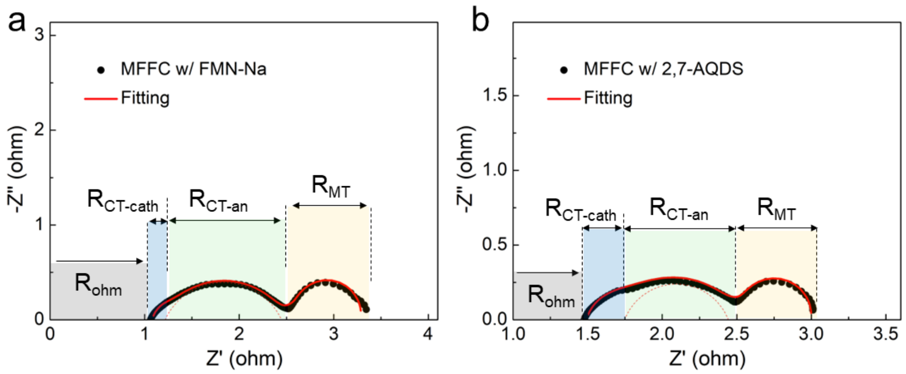

Supplementary Figure 17 | The EIS measurements of MFFC systems. a, EIS measurement after multiple performance tests of the MFFC system with adding FMN-Na into the bacteria anolyte. **b**, EIS measurement of the MFFC system with adding 2,7-AQDS into the bacteria anolyte. The resistance (R_{ohm}, R_{CT} and R_{MT}) values are listed in Supplementary Table 3.

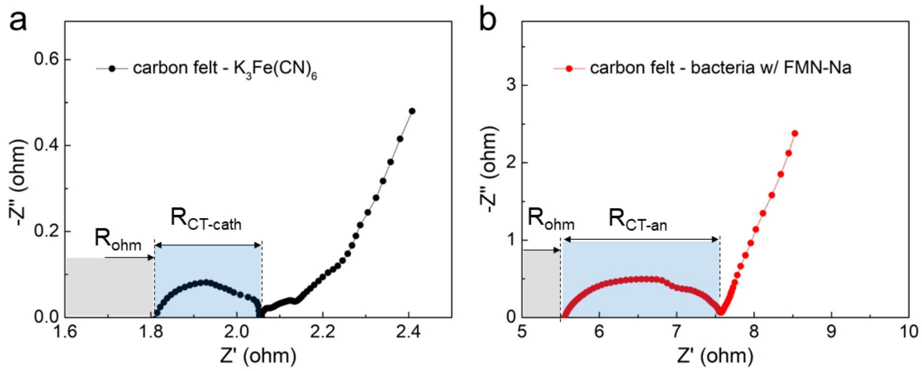

Supplementary Figure 18 | The EIS measurements of the carbon felt electrode in different solutions with the three-electrode setup. a, EIS measurement of the carbon felt electrode in the ferricyanide catholyte. **b**, EIS measurement of the carbon felt electrode in the bacteria anolyte with adding FMN-Na. The resistance (R_{ohm} and R_{CT}) values are listed in Supplementary Table 3.

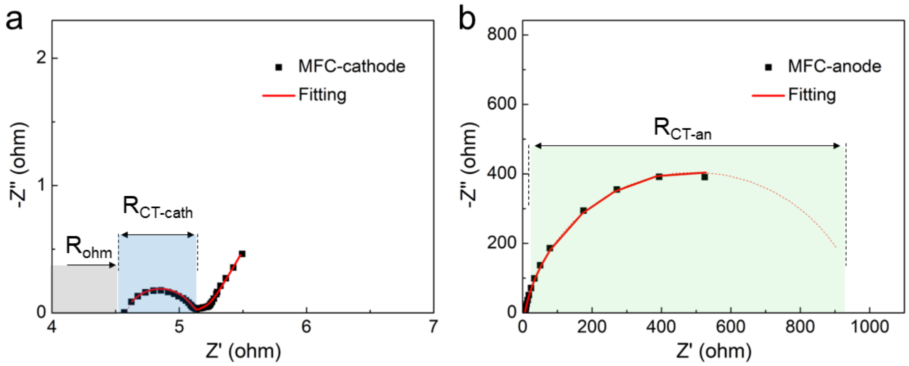

Supplementary Figure 19 | Internal resistance analysis of MFC systems. a, EIS measurement of MFC cathode (ferricyanide) with using the carbon cloth based on the three-electrode setup. **b**, EIS measurement of MFC anode with using the carbon felt after incubation based on the three-electrode setup. The values of R_{ohm} and R_{CT} are listed in Supplementary Table 3.

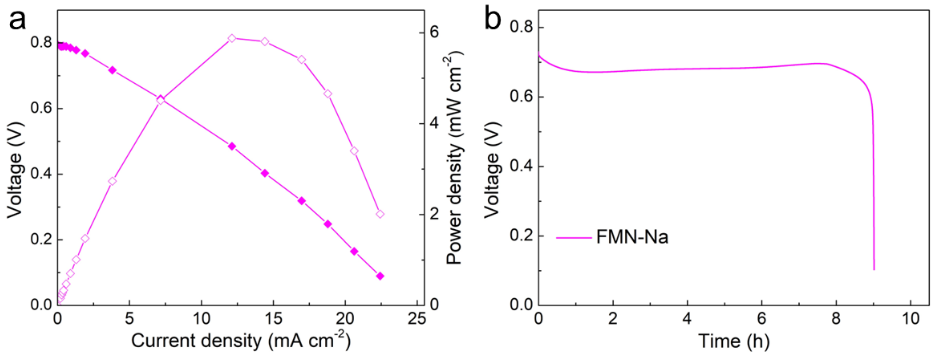

Supplementary Figure 20 | Performance of the redox-mediated MFFCs with the synthetic wastewater. **a**, Polarization curves and power output of the FMN-Na-mediated MFFCs using the synthetic wastewater. **b**, Long-term operation of the FMN-Na-mediated MFFCs using the synthetic wastewater (120 ml) at a constant current of 3 mA cm^{-2}.

Supplementary Table 1 | Comparison of current and power density of conventional MFCs and mediated MFFCs.

Bacteria type	Redox mediator	Anode	Cathode	Current density (mA cm^{-2})	Power density (mW cm^{-2})
Mixed culture[5]	/	graphite carbon brush	ferricyanide	0.85	0.246
Geobacter spp[6]	/	carbon felt (flow)	oxygen	5	0.88
Geobacter sulfurreducens[7]	/	MWCNT/Chitosan	oxygen	1.9	0.287
Geobacter sulfurreducens[8]	/	graphite cloth	ferricyanide	0.5	0.16
Geobacter[9]	/	vertically aligned CNT	ferricyanide	0.26	0.083
Mixed culture[10]	/	NCP/LSC	oxygen	0.5	0.11
P. aeruginosa[11]	/	VSG/chitosan	ferricyanide	0.55	0.153
E. Coli DH5α[12]	/	PBE/CNT/carbon felt	oxygen	1.6	0.38
Rhodopseudomonas palustris DX-1[13]	/	graphite fiber brush	oxygen	0.99	0.27
Shewanella putrefacians[14]	/	ILP/carbon felt	ferricyanide	2.3	0.44
Mixed culture[15]	/	carbon paper	ferricyanide	0.04	0.035
Mixed culture[16]	/	carbon felt (flow)	oxygen	4.5	0.71
Mixed culture[17]	/	carbon fiber cloth	oxygen	2.6	0.686
Mixed culture[18]	/	rGO/MnO_2/CF (flow)	ferricyanide	0.9	0.21
Mixed culture[19]	/	graphene sponge	oxygen	0.132	0.157
Shewanella MR-1[20]	/	3D graphene/PANI	ferricyanide	0.45	0.077
Shewanella DSP10[21]	/	graphite felt (flow)	ferricyanide	0.4	0.2
Shewanella MR-1[22]	/	rGO/Pt	ferricyanide	0.69	0.148
Shewanella MR-1[23]	/	rGO/Ag/carbon paper	oxygen	3.85	0.66
Shewanella DSP10[24]	2,6-AQDS	graphite felt (flow)	ferricyanide	1.8	0.4
S. cerevisiae[25]	9,10-PQ	graphite	ferricyanide	0.58	0.0022
Shewanella MR-1[26]	RF	CF/GO-MWCNT/PRF	ferricyanide	0.8	0.37
Mixed culture[27]	2-AQS	CF/PPy/AQS	oxygen	1	0.192
Mixed culture[28]	RF	Graphite felt	oxygen	0.1	0.01
Shewanella S12[29]	2,6-AQDS	CF/PPy/AQDS	ferricyanide	0.36	0.13
Shewanella MR-1[30]	RF	RF/graphene/GP	ferricyanide	0.3	0.0257
Shewanella MR-1	2,7-AQDS	CF (MFFC, this work)	ferricyanide	42	13.1
Shewanella MR-1	FMN-Na	CF (MFFC, this work)	ferricyanide	33	10.2

*The mixed culture means more than two kinds of unspecified bacteria used in the system. NCP/LSC: nitrogen-enriched carbon nanoparticle-modified loofah sponge carbon; VSG: vacuum stripped graphene; PBE: Poly(bisphenol A-co-epichlorohydrin); ILP: ionic liquid polymer; 2,6-AQDS: anthraquinone-2,6-disulphonic disodium; 2-AQS: Anthraquinone-2-sulfonate; 9,10-PQ: 9,10-phenantrenequinone; RF: riboflavin; CF: carbon felt.

Supplementary Table 2 | Comparison of current and power density with or without redox mediators.

Bacteria anode	Redox mediator	Cathode	Current density with (without) redox mediators (mA cm^{-2})	Power density with (without) redox mediators (mW cm^{-2})
Shewanella DSP10[24]	2,6-AQDS	ferricyanide	1.8 (1.0)	0.4 (0.3)
S. cerevisiae[25]	9,10-PQ	ferricyanide	0.58 (N.A.)	0.0022 (N.A.)
Shewanella MR-1[26]	RF	ferricyanide	0.80 (0.32)	0.37 (0.12)
Mixed culture[27]	2-AQS	oxygen	1.0 (0.48)	0.192 (0.072)
Mixed culture[28]	RF	oxygen	0.1 (0.05)	0.01 (0.0052)
Shewanella S12[29]	2,6-AQDS	ferricyanide	0.36 (0.13)	0.13 (0.01)
Shewanella MR-1[30]	RF	ferricyanide	0.3 (0.1)	0.0257 (0.0075)
Shewanella MR-1 (this work)	2,7-AQDS	ferricyanide	**42 (0.7)**	**13.1 (0.19)**
Shewanella MR-1 (this work)	FMN-Na	ferricyanide	**33 (0.7)**	**10.2 (0.19)**

2,6-AQDS: anthraquinone-2,6-disulphonic disodium; 2-AQS: Anthraquinone-2-sulfonate; 9,10-PQ: 9,10-phenantrenequinone; RF: riboflavin.

Supplementary Table 3 | The resistance values determined from the three-electrode, MFFC, and MFC EIS tests.

Three-electrode	MFC-cathode	MFC-anode	CF-ferricyanide	CF-bacteria w/ FMN-Na
R_{ohm} (Ω)	4.6	6.4	1.8	5.5
R_{CT} (Ω)	0.54	974.1	0.23	2.1

MFC or MFFC	MFC	MFFC	MFFC w/ FMN-Na
R_{ohm} (Ω)	41.5	0.91	0.76
$R_{CT\text{-}cath}$ (Ω)	0.57	0.27	0.16
$R_{CT\text{-}an}$ (Ω)	978.3	280.8	0.70
R_{MT} (Ω)	/	/	0.70

MFFC	MFFC w/ FMN-Na (after multiple test)	MFFC w/ 2,7-AQDS
R_{ohm} (Ω)	1.08	1.49
$R_{CT\text{-}cath}$ (Ω)	0.15	0.26
$R_{CT\text{-}an}$ (Ω)	1.23	0.70
R_{MT} (Ω)	0.84	0.56

Note: CF represents the carbon felt electrode.

References

1 Huskinson, B. *et al.* A metal-free organic–inorganic aqueous flow battery. *Nature* **505**, 195-198 (2014).

2 Wedege, K., Dražević, E., Konya, D. & Bentien, A. Organic Redox Species in Aqueous Flow Batteries: Redox Potentials, Chemical Stability and Solubility. *Sci. Rep.* **6**, 39101 (2016).

3 Orita, A., Verde, M. G., Sakai, M. & Meng, Y. S. A biomimetic redox flow battery based on flavin mononucleotide. *Nat. Commun.* **7**, 1-8 (2016).

4 Wiberg, C., Carney, T. J., Brushett, F., Ahlberg, E. & Wang, E. Dimerization of 9,10-anthraquinone-2,7-Disulfonic acid (AQDS). *Electrochim. Acta* **317**, 478-485 (2019).

5 Lawson, K., Rossi, R., Regan, J. M. & Logan, B. E. Impact of cathodic electron acceptor on microbial fuel cell internal resistance. *Bioresour. Technol.* **316**, 123919 (2020).

6 Rossi, R. & Logan, B. E. Using an anion exchange membrane for effective hydroxide ion transport enables high power densities in microbial fuel cells. *Chem. Eng. J.* **422**, 130150 (2021).

7 Katuri, K. *et al.* Three-dimensional microchanelled electrodes in flow-through configuration for bioanode formation and current generation. *Energy Environ. Sci.* **4**, 4201-4210 (2011).

8 Nevin, K. P. *et al.* Power output and columbic efficiencies from biofilms of Geobacter sulfurreducens comparable to mixed community microbial fuel cells. *Environ. Microbiol.* **10**, 2505-2514 (2008).

9 Ren, H. *et al.* A high power density miniaturized microbial fuel cell having carbon nanotube anodes. *J. Power Sources* **273**, 823-830 (2015).

10 Yuan, Y., Zhou, S., Liu, Y. & Tang, J. Nanostructured Macroporous Bioanode Based on Polyaniline-Modified Natural Loofah Sponge for High-Performance Microbial Fuel Cells. *Environ. Sci. Technol.* **47**, 14525-14532 (2013).

11 He, Z., Liu, J., Qiao, Y., Li, C. M. & Tan, T. T. Y. Architecture Engineering of Hierarchically Porous Chitosan/Vacuum-Stripped Graphene Scaffold as Bioanode for High Performance Microbial Fuel Cell. *Nano Lett.* **12**, 4738-4741 (2012).

12 Li, H. *et al.* Power output of microbial fuel cell emphasizing interaction of anodic binder with bacteria. *J. Power Sources* **379**, 115-122 (2018).

13 Xing, D., Zuo, Y., Cheng, S., Regan, J. M. & Logan, B. E. Electricity Generation by Rhodopseudomonas palustris DX-1. *Environ. Sci. Technol.* **42**, 4146-4151 (2008).

14 Yang, L. *et al.* Boosting current generation in microbial fuel cells by an order of magnitude by coating an ionic liquid polymer on carbon anodes. *Biosens. Bioelectron.* **91**, 644-649 (2017).

15 Oh, S.-E. & Logan, B. E. Proton exchange membrane and electrode surface areas as factors that affect power generation in microbial fuel cells. *Appl. Microbiol. Biotechnol.* **70**, 162-169 (2006).

16 Rossi, R., Wang, X. & Logan, B. E. High performance flow through microbial fuel cells with anion exchange membrane. *J. Power Sources* **475**, 228633 (2020).

17 Fan, Y., Sharbrough, E. & Liu, H. Quantification of the Internal Resistance Distribution of Microbial Fuel Cells. *Environ. Sci. Technol.* **42**, 8101-8107 (2008).

18 Zhang, C. *et al.* Binder-free graphene and manganese oxide coated carbon felt anode for high-performance microbial fuel cell. *Biosens. Bioelectron.* **81**, 32-38 (2016).

19 Xie, X. *et al.* Graphene–sponges as high-performance low-cost anodes for microbial fuel cells. *Energy Environ. Sci.* **5**, 6862-6866 (2012).

20 Yong, Y.-C., Dong, X.-C., Chan-Park, M. B., Song, H. & Chen, P. Macroporous and Monolithic Anode Based on Polyaniline Hybridized Three-Dimensional Graphene for High-Performance Microbial Fuel Cells. *ACS Nano* **6**, 2394-2400 (2012).

21 Ringeisen, B. R., Ray, R. & Little, B. A miniature microbial fuel cell operating with an aerobic anode chamber. *J. Power Sources* **165**, 591-597 (2007).

22 Zhao, S. *et al.* Three-dimensional graphene/Pt nanoparticle composites as freestanding anode for enhancing performance of microbial fuel cells. *Sci. Adv.* **1**, e1500372 (2015).

23 Cao, B. *et al.* Silver nanoparticles boost charge-extraction efficiency in Shewanella microbial fuel cells. *Science* **373**, 1336-1340 (2021).

24 Ringeisen, B. R. *et al.* High Power Density from a Miniature Microbial Fuel Cell Using Shewanella oneidensis DSP10. *Environ. Sci. Technol.* **40**, 2629-2634 (2006).

25 Rozene, J., Morkvenaite-Vilkonciene, I., Bruzaite, I., Dzedzickis, A. & Ramanavicius, A. Yeast-based microbial biofuel cell mediated by 9, 10-phenantrenequinone. *Electrochim. Acta* **373**, 137918 (2021).

26 Zhao, J. *et al.* Elongated Riboflavin-Producing Shewanella oneidensis in a Hybrid Biofilm Boosts Extracellular Electron Transfer. *Adv. Sci.* **10**, 2206622 (2023).

27 Tang, X. & Ng, H. Y. Anthraquinone-2-sulfonate immobilized to conductive polypyrrole hydrogel as a bioanode to enhance power production in microbial fuel cell. *Bioresour. Technol.* **244**, 452-455 (2017).

28 Sun, J., Li, W., Li, Y., Hu, Y. & Zhang, Y. Redox mediator enhanced simultaneous decolorization of azo dye and bioelectricity generation in air-cathode microbial fuel cell. *Bioresour. Technol.* **142**, 407-414 (2013).

29 Feng, C. *et al.* A polypyrrole/anthraquinone-2, 6-disulphonic disodium salt (PPy/AQDS)-modified anode to improve performance of microbial fuel cells. *Biosens. Bioelectron.* **25**, 1516-1520 (2010).

30 Wang, Q.-Q. *et al.* Facile in-situ fabrication of graphene/riboflavin electrode for microbial fuel cells. *Electrochim. Acta* **232**, 439-444 (2017).
